# Bridging potential and achievement: the AI-enhanced Latin American model of talent development

**DOI:** 10.3389/fpsyg.2026.1988238

**Published:** 2026-10-14

**Authors:** Sheyla Blumen, Eduardo Franco, Keytha Alcantara, Arianna Benavides

**Affiliations:** Departamento Académico de Psicología, Pontificia Universidad Católica del Perú, Lima, Peru

**Keywords:** artificial intelligence, equity, gifted education, human agency, personalized learning, talent development, twice-exceptionality, underachievement

## Abstract

The persistent gap between individual potential and realized achievement remains a central challenge in gifted education and talent development, particularly when strengths are unrecognized or opportunities are constrained. This Hypothesis and Theory article introduces the AI-Enhanced Latin American Model of Talent Development (AI-LAMTD), an ecological and equity-centered framework that examines underachievement through dynamic potential-opportunity mismatch. Potential is a provisional inference about further domain development under specified conditions; giftedness or high ability describes unusually advanced capacity relative to an explicit reference context; competence comprises acquired domain knowledge and skills; talent is outstanding developed domain competence; and achievement is an observable accomplishment at a particular time. These constructs serve different explanatory and measurement roles. The framework retains four interacting ecological levels—individual development, learning contexts, family and community, and policy and infrastructure—within the cross-cutting principles of equity and capability expansion and cultural relevance and inclusion. Five developmental functions organize possible AI support: recognition of emerging potential, opportunity design, engagement and feedback, developmental monitoring, and support for well-being and flourishing. Recognition, opportunity conversion, engagement, learning and practice, and independent transfer are recurrent conversion processes through which domain competence may develop into talent. Potential is reassessed throughout development, while achievement, agency, well-being, and contribution are evaluated over time; flourishing is not an automatic consequence of advanced competence. Ten falsifiable propositions specify relationships, boundary conditions, comparisons, and outcomes concerning recognition, opportunity conversion, independent development, and equity. Evidence from general AI in education informs mechanism plausibility and is distinguished from the emerging evidence specific to gifted and high-ability learners. AI is neither necessary nor sufficient for talent development. Its developmental value depends on accessible opportunity, learner agency, well-being, and competence that persists beyond the focal technological assistance.

## 1 Introduction

One of the central challenges of educational psychology is to explain why emerging capacity becomes sustained accomplishment for some learners but remains unrealized for others. Major talent-development traditions address this question from different theoretical positions. Gagné’s Differentiated Model of Giftedness and Talent distinguishes natural abilities from systematically developed competencies and identifies intrapersonal and environmental catalysts (Gagné, 2004). Subotnik et al. (2011) conceptualize giftedness developmentally, with domain-specific competence, expertise, and eminent performance becoming increasingly central across developmental stages. The enrichment tradition emphasizes opportunities for advanced, interest-driven learning and creative productivity (Reis and Renzulli, 2010; Reis et al., 2021). The Actiotope Model locates gifted action within an adaptive learner-environment system comprising goals, action repertoires, subjective action space, and environmental resources (Ziegler, 2005; Ziegler et al., 2013). These traditions overlap in treating achievement as developmentally produced, but they are not theoretically interchangeable.

AI-LAMTD adopts the following working definitions. Potential is a provisional inference about a learner’s capacity for further development in a specified domain under stated opportunity and support conditions. It is inferred from converging evidence, including learning rate, responsiveness to challenge or scaffolding, transfer, domain reasoning, and creative production. Potential does not itself imply exceptional standing and remains relevant after advanced competence has developed. Giftedness or high ability describes capacity judged to be unusually advanced relative to an explicit developmental, domain, and reference context, supported by demonstrated ability or credible evidence of advanced learning capacity. Thus, potential concerns what may develop under particular conditions, whereas high ability adds a comparative judgment about the level of capacity. Recognition is the evidence-based decision process through which such strengths are identified; it is distinct from the capacity being inferred.

Competence refers to acquired knowledge and skills that support effective action within a domain across relevant tasks. Talent is outstanding, systematically developed domain competence, evidenced repeatedly against explicit domain standards or an appropriate reference group. Competence therefore admits a range of levels, whereas talent specifies a distinguished level of developed competence. Expertise refers to advanced mastery within a domain and may characterize developed talent; the terms are not used as interchangeable stage labels in AI-LAMTD. Achievement is an observable accomplishment at a specified time, such as a test result, performance, or product. It supplies evidence about competence but also depends on task demands, opportunity, and assistance. Immediate AI-assisted achievement is therefore measured separately from retained competence and independent transfer.

These are model-specific definitions rather than a claim that the contributing traditions share one taxonomy. AI-LAMTD adopts Gagné’s distinction between ability and systematically developed competence while requiring studies to state their own justified reference groups and thresholds; it does not import a universal percentile rule or equate potential with natural ability (Gagné, 2004). Subotnik et al. (2011) inform the increasing developmental importance of demonstrated domain performance. Enrichment approaches inform opportunities for interest-driven learning and creative production, and the Actiotope Model situates evidence of capacity and competence within an adaptive learner-environment system (Reis and Renzulli, 2010; Reis et al., 2021; Ziegler, 2005; Ziegler et al., 2013). Table 1 specifies operational distinctions. Each study should prespecify its domain, reference context, assessment conditions, and decision criteria, examine their local validity, and report uncertainty. The framework does not require a giftedness label before an appropriate developmental opportunity can be offered.

**Table 1:** Conceptual distinctions and illustrative operational indicators.

Construct	Conceptual and operational distinction	Illustrative indicators and assessment conditions
Potential	Provisional inference about further development in a specified domain under stated opportunities and support. It does not itself denote exceptional standing.	Converging learning-rate, scaffold-response, transfer, reasoning, and creative-production evidence; repeated assessment with uncertainty reported.
Giftedness or high ability	Unusually advanced capacity relative to an explicit domain and developmental reference context. A comparative description, distinct from the recognition decision.	Convergent ability and dynamic-learning evidence; stated reference group and justified criteria; local validity, subgroup error analysis, and revisability.
Competence	Acquired domain knowledge and skills demonstrated across relevant tasks. Distinct from the subjective experience of competence in self-determination theory.	Domain-aligned task battery or rubric; retention, generalization, and transfer; assistance and accessibility accommodations recorded.
Talent	Outstanding, systematically developed domain competence. Talent specifies a distinguished level of competence, rather than any acquired proficiency.	Repeated advanced performances or products evaluated against explicit domain standards or a justified reference group; evidence of independent retention and transfer.
Achievement	Observable accomplishment on a task, product, or performance at a specified time. Evidence about competence, not an exhaustive measure of it.	Scores, grades, products, or assessed performances; task demands, opportunity, timing, and AI or human assistance documented.
Recognition	A warranted, revisable judgment that evidence indicates a strength requiring differentiated opportunity. The decision process is distinct from inferred capacity.	Multi-source decisions; confidence estimates; stated local norms; decision stability and revision; sensitivity and specificity against independent later criteria.
Developmental opportunity	Accessible, appropriately challenging, sustained provision. Opportunity conversion concerns whether resources become provision that can be taken up and sustained.	Access, uptake, duration, intensity, challenge, mentor quality, continuity, and learner-opportunity fit.
Underachievement	Persistent or recurrent shortfall from warranted developmental expectations, investigated through potential-opportunity mismatch across conversion processes.	Prespecified longitudinal shortfall from developmental expectations; outcome measured independently of explanatory opportunity indicators; alternative explanations and learner goals examined.
Independent transfer	Application or adaptation of learned competence beyond the original task and without the focal AI assistance. Necessary access accommodations are retained.	New-task and new-context performance; delayed assessments; scaffold fading; explicit record of retained accommodations and removed assistance.
Agency	Capacity to formulate goals, make meaningful choices, regulate learning, and evaluate or contest recommendations.	Goal authorship, strategic choice, help seeking, self-monitoring, justified rejection of AI advice, and reduced dependence on focal scaffolds.
Cultural relevance	Alignment of constructs, evidence, opportunities, and valued outcomes with learners’ languages, identities, knowledge systems, and communities.	Local content validity, language accessibility, measurement invariance where relevant, stakeholder judgments, and culturally meaningful products.
Flourishing	Sustained competence together with well-being, agency, belonging, and meaningful contribution. An evaluative outcome, not an inevitable stage after talent.	Separate longitudinal measures of competence, well-being, agency, belonging, engagement, and contribution valued by learners and communities.

Underachievement is traditionally defined as a discrepancy between potential or ability and actual achievement. This discrepancy describes a shortfall from expectations but does not identify its causes. A systematic review identified emotional, social, family, socioeconomic, cultural, and educational factors associated with gifted underachievement (Raoof et al., 2024). A broader meta-analysis linked underachievement to lower competence beliefs, mastery goals, autonomous motivation, task values, and self-regulated learning strategies (Fong et al., 2023). A meta-analysis of interventions for gifted underachievement found no statistically significant overall academic benefit, alongside small positive psychosocial effects; the authors emphasized the limited quality of the available evidence (Steenbergen-Hu et al., 2020). These findings motivate closer investigation of developmental mechanisms but do not establish that existing interventions are generally ineffective.

This problem is especially evident for twice-exceptional (2e) learners. Their high potential can compensate for disability-related challenges, while disability can suppress the behavioral expression of their strength; the two may mask one another and produce seemingly average or inconsistent performance (Baum et al., 2014; Beckmann and Minnaert, 2018; Foley Nicpon et al., 2011; Reis et al., 2014; Rizzo et al., 2025). Strength-focused programming can therefore be withheld from students with profiles that make achievement an unreliable proxy for potential. This masking problem makes it an important test for any technology-based identification system: an algorithm trained to associate giftedness with uniformly high achievement may overlook the learners for whom dynamic, multidimensional evidence is most needed.

As AI development accelerates, the theoretical problem becomes more pressing. Artificial intelligence in education now encompasses intelligent tutoring, learner modeling, automated and dynamic assessment, adaptive learning, learning analytics, generative AI, and digital mentoring. Reviews document expanding applications alongside persistent concerns about pedagogy, teacher roles, evidence quality, fairness, and the adverse effects of optimizing narrow outcome variables (Zawacki-Richter et al., 2019; Chen et al., 2020; Ouyang and Jiao, 2021; Holmes et al., 2022; Garzón et al., 2025). In gifted and talented education specifically, a 2026 scoping review identified an emerging literature on differentiation, mentoring, creativity, assessment, gifted identification, and twice-exceptional support, while emphasizing the need for longitudinal, comparative, and equity-centered research (Alsamani and Alsamiri, 2026). The central argument of this article is therefore not that AI can solve underachievement. Rather, AI can become developmentally useful when it helps educational systems observe and respond to mismatches between potential and opportunity. This distinction is especially important because technology can enhance immediate task performance without improving independent human capability. In a field experiment in high-school mathematics, unrestricted generative AI substantially improved performance while it was available, but students performed worse than controls when AI access was subsequently removed, illustrating that assisted performance cannot automatically be interpreted as independent learning (Bastani et al., 2025).

Existing talent-development and ecological models establish that abilities develop through interactions with opportunities, motivation, relationships, and context. Building on these accounts, AI-LAMTD focuses on a specific integrative problem: locating disruptions in development, specifying how information about learners is converted into opportunity, and testing how algorithmic mediation changes these processes. Similar observed achievement gaps may arise through different mechanisms and therefore require different interventions. The value of the proposed integration must be established through comparison with existing explanations.

AI-LAMTD addresses this problem through three theoretical additions to LAMTD. First, it treats potential-opportunity mismatch as a changing unit of explanation located in recurrent conversion processes: recognition, opportunity conversion, engagement, learning and practice, and independent transfer. Second, it conceptualizes AI-mediated classification, recommendation, adaptation, feedback, and monitoring as components of developmental infrastructure that may alter these processes in beneficial, null, or harmful directions. Third, it derives conditional propositions specifying when mismatch should occur, which mechanism should mediate or moderate it, and which observations would fail to support the prediction. Developmental transitions refer to changes in domain competence over time; conversion processes help explain whether and how those changes occur.

The relevant question is therefore whether, for whom, and under which ecological conditions AI-supported systems promote independently sustainable competence, agency, well-being, and contribution. The present Hypothesis and Theory article develops ten propositions that distinguish immediate assisted performance from development that persists beyond technological support and that identify conditions under which AI may reduce, reproduce, or intensify potential-opportunity mismatch.

## 2 From discrepancy to ecological mismatch

A discrepancy between potential and achievement is an outcome, not a mechanism. When a high-ability student receives low grades, common explanations focus on motivation, self-regulation, perfectionism, attention, or effort. These factors matter, but self-regulated learning research makes clear that regulation is cyclical, contextual, and responsive to task conditions, goals, feedback, and perceived control (Panadero, 2017). Feedback research likewise demonstrates that the information learners receive can redirect goals, strategies, self-monitoring, and subsequent performance, depending on its timing, specificity, and level (Hattie and Timperley, 2007; Shute, 2008). An ecological explanation therefore asks how learner characteristics and environmental conditions interact rather than if low achievement reveals an internal failure to actualize ability.

Bronfenbrenner and Morris’s (2006) bioecological model provides a useful foundation because it treats development as the product of reciprocal interactions among person, process, context, and time. Applied to talent development, the relevant developmental unit is not the learner in isolation but the learner-in-context. This ecological perspective is further specified by Ziegler’s Actiotope Model of Giftedness, which conceptualizes talent development not as the unfolding of a relatively fixed individual attribute, but as a dynamic process occurring within an actiotope, the system constituted by an individual and the material, social, and informational environments with which that individual interacts. From this perspective, successful talent development depends on the progressive coordination and adaptation of the learner’s action repertoire, subjective action space, goals, and environmental resources, as well as on the capacity of the overall system to respond effectively to changing developmental demands (Ziegler, 2005; Ziegler et al., 2013). A student’s opportunities to encounter advanced content, skilled teachers, meaningful mentors, culturally responsive instruction, psychological support, and sustained challenge therefore influence not only whether potential can be converted into increasingly differentiated competence, but also whether the learner–environment system can develop the resources and adaptive pathways necessary to sustain that development over time. Talent-development theories converge on this point from different directions: Gagné (2004) distinguishes natural abilities from systematically developed talents and identifies intrapersonal and environmental catalysts; Subotnik et al. (2011) describe a developmental progression from potential to competence and expertise; and the Schoolwide Enrichment Model emphasizes access to enriched, interest-driven opportunities and creative productivity rather than identification as an endpoint (Reis and Peters, 2021; Reis et al., 2021).

AI-LAMTD examines underachievement as a persistent or recurrent shortfall from warranted developmental expectations associated with potential-opportunity mismatch. Its contribution is to locate and track the processes through which such a shortfall may arise. A recognition mismatch occurs when evidence of emerging capacity remains invisible or is misclassified. An opportunity mismatch occurs when recognized strengths are not met with accessible and appropriately challenging provision. An engagement mismatch occurs when available opportunities do not support autonomy, perceived competence, relatedness, belonging, identity, or meaningful goals. A support mismatch occurs when disability-related needs, emotional distress, language barriers, executive-function challenges, or economic constraints obstruct participation or learning. A systemic mismatch occurs when policy, institutional capacity, infrastructure, or governance constrains multiple conversion processes. These forms may coexist and change over time. A mismatch signal indicates a hypothesis for investigation; it does not by itself establish underachievement or justify overriding a learner’s autonomous goals.

This account generates different intervention predictions for learners with similar achievement discrepancies. Unrecognized learning responsiveness directs attention to recognition; recognition without advanced provision directs attention to opportunity conversion; participation without retained learning or transfer directs attention to learning and practice and independent transfer. Achievement gains accompanied by declining well-being require evaluation of support and outcome quality. These are diagnostic hypotheses to be tested against alternatives using longitudinal, multi-source evidence. Recognition, participation, and transfer can each be revisited during competence development, rather than occurring once in a fixed sequence.

*Proposition 1*: Ecological mismatch. Among learners with comparable baseline indicators of emerging potential, longitudinal underachievement will be predicted more accurately by learner-by-opportunity fit—including access, challenge, support, and continuity—than by learner characteristics or a cross-sectional potential-achievement discrepancy alone. This proposition would not be supported if interaction terms and opportunity indicators fail to add out-of-sample predictive value beyond learner-only and discrepancy-only models.

## 3 Why mismatch is unequally distributed

Identification systems can amplify pre-existing inequality because prior access to enriched learning influences the scores, grades, teacher judgments, and portfolios later treated as evidence of potential. Evidence from universal screening and local norms in the United States shows that identification architecture changes who becomes visible (Card and Giuliano, 2016; Peters et al., 2019, 2021). These findings show that screening and norming procedures affect representation in the systems studied; whether the same mechanisms and effect sizes occur elsewhere is an empirical question requiring locally valid indicators and cross-national testing.

Latin American evidence makes the relevance of opportunity conditions visible. ERCE 2019 assessed approximately 160,000 primary-school students in 16 countries and reported learning differences associated with socioeconomic background, school location, and educational context (UNESCO Regional Office for Education in Latin America and the Caribbean, 2021). A regional report documents inequalities in connectivity and digital skills, including disadvantages affecting rural and lower-income households (Economic Commission for Latin America and the Caribbean, 2022). These findings do not establish a uniform form of potential-opportunity mismatch across countries. They motivate examining recognition, recommendation, access, and sustained participation separately.

Latin America is linguistically, culturally, and institutionally heterogeneous. Indigenous and migrant learners may move among home languages, national languages, and school-based academic registers. Assessment instruments, teacher expectations, or AI models that treat one linguistic repertoire as neutral may confuse cultural or linguistic distance with limited potential. Rurality may add constraints where advanced coursework, specialist services, and mentors are geographically concentrated. Gifted and talent-development provision likewise varies among and within countries: formal recognition may coexist with uneven implementation, limited professional preparation, centralized services, or weak continuity between identification and sustained programming (Blumen, 2026). This variation provides testable contrasts in the institutional conditions that convert recognition into development.

Twice-exceptionality further illustrates why achievement may be an incomplete proxy for potential. Reviews document masking, adverse school experiences, and the need to model advanced strengths and disability-related barriers together (Foley Nicpon et al., 2011; Reis et al., 2014; Beckmann and Minnaert, 2018; Rizzo et al., 2025). AI systems may help integrate longitudinal and multidimensional evidence, but historical labels, underrepresented languages, and unequal error rates may reproduce prior exclusion (Barocas and Selbst, 2016; Blodgett et al., 2020; Mehrabi et al., 2021). The capability approach therefore shifts evaluation from nominal resource availability to substantive opportunity: who can use a resource, under which conversion conditions, and toward which learner- and community-valued forms of development (Sen, 1999; Nussbaum, 2011).

## 4 AI as developmental infrastructure

AI-LAMTD treats AI not merely as a set of instructional tools but as potential developmental infrastructure. When technology influences how learners are identified, assigned challenge, provided feedback, monitored, mentored, and connected to resources, it becomes part of the ecology through which potential is converted into achievement.

### 4.1 Define AI by technology and function

In this article, AI is an umbrella category, not a unitary intervention. The framework distinguishes technologies by the developmental function they may serve and by the evidence and risks attached to each use. Pattern detection and learner modeling may inform recognition or monitoring; dynamic assessment and process analytics may estimate learning rate, transfer, strategy change, or responsiveness to scaffolding; adaptive sequencing and recommender systems may alter challenge, pace, or access to resources; intelligent tutoring and generative systems may provide hints, explanations, feedback, or metacognitive prompts; digital mentoring and resource-matching systems may connect learners with people, programs, and advanced opportunities; and longitudinal learning analytics may detect growth, plateau, or disengagement. These applications differ in construct validity, data requirements, evidence base, and ethical risk.

AI-LAMTD therefore assigns no general developmental effect to “AI”; effects are conditional on the technology, pedagogical design, target construct, user population, and surrounding ecology. Each empirical study should identify the capability, the developmental function, the target process, and the human oversight arrangement. The same technology may serve different functions, and a function may be implemented without AI (see Table 2).

**Table 2:** AI capabilities, developmental functions, and principal risks.

AI capability	Typical mechanism	Primary developmental function	Key developmental risk
Pattern detection and learner modeling	Infer trajectories, profiles, or latent states from performance and process data	Recognition of emerging potential; developmental monitoring	Historical labels, construct underrepresentation, identity assignment
Dynamic assessment and process analytics	Estimate learning rate, transfer, strategy change, and response to scaffolding	Recognition of emerging potential; developmental monitoring	Intrusive measurement, invalid proxies, unequal error rates
Adaptive sequencing and recommendation	Adjust challenge, pace, resources, and next learning opportunities	Opportunity design	Remediation-only adaptation, restricted exploration, inaccessible recommendations
Intelligent tutoring and generative feedback	Provide hints, explanations, feedback, prompts, and guided practice	Engagement and feedback	Answer substitution, overreliance, hallucination, weakened self-regulation
Digital mentoring and resource matching	Connect learners with people, courses, communities, and advanced resources	Opportunity design; support for well-being and flourishing	Poor fit, unsafe interaction, exclusion through connectivity or cost
Longitudinal learning analytics	Detect growth, plateau, disengagement, or emerging mismatch over time	Developmental monitoring	Surveillance, false alerts, institutional inability to respond

Research on adaptive systems illustrates both promise and boundary conditions. A meta-analysis of 45 studies found a medium-to-large average advantage for AI-enabled adaptive learning over non-adaptive interventions on cognitive outcomes, with variation across learners, disciplines, duration, design, and adaptation features (Wang et al., 2024). A review of K-12 intelligent tutoring systems found generally positive effects, but smaller advantages when compared with non-intelligent tutoring systems and a need for longer, more diverse studies (Létourneau et al., 2025). Zhang (2026) illustrates learner-state modeling and the distinction between short- and longer-horizon prediction. That study used offline benchmarks and computational proxies; it does not establish causal classroom effects or validated measurement of developmental potential.

### 4.2 Distinguish direct and indirect evidence

The evidentiary status of these claims must be differentiated. Research conducted with gifted, high-ability, or twice-exceptional learners constitutes direct population-relevant evidence. Studies of broader student populations provide indirect evidence for mechanism plausibility—for example, that an adaptive system can change practice conditions or that scaffolded prompts can influence self-regulation—but do not establish equivalent effects, validity, or equity for advanced learners. Gifted-education-specific AI research remains emerging (Alsamani and Alsamiri, 2026). Accordingly, the applications proposed here are hypotheses for targeted validation, not conclusions that general AIEd findings automatically generalize to gifted education.

The first AI-enabled function in AI-LAMTD is recognition of emerging potential. Static assessment measures what a learner demonstrates at a particular time under specified conditions; developmental assessment asks how quickly the learner acquires, transfers, reorganizes, or generalizes knowledge under appropriately scaffolded challenge. Learning analytics can extend this logic by examining trajectories rather than single scores. Roll and Winne (2015) argued that learning analytics can be used to understand, evaluate, and support self-regulated learning, while Cloude et al. (2022) described multimodal trace data architectures capable of representing cognitive, affective, metacognitive, and motivational processes. A systematic review of multimodal SRL research now shows movement from unimodal measures toward integration of log, physiological, behavioral, and other data streams, although motivation and affect remain comparatively underrepresented (de Mooij et al., 2025). These developments motivate Proposition 3, which remains to be tested in the target populations: learning rate, transfer, responsiveness to scaffolding, and strategy use should add information about emerging potential beyond static achievement, especially when prior opportunities have been unequal.

The second function is opportunity design. Recognition without provision is developmentally weak: a label does not teach, mentor, enrich, accelerate, or create expertise. The Schoolwide Enrichment Model and related enrichment research emphasize that advanced learners require sufficiently complex, interest-based, and productive opportunities, not simply more of the standard curriculum (Reis and Peters, 2021; Reis et al., 2021). AI-supported personalization may help match challenge, pacing, resources, mentors, and creative tasks to learner profiles. A system that adapts only toward a fixed proficiency threshold may fail to extend advanced strengths. Talent development requires upward adaptation—continued movement beyond proficiency toward depth, complexity, creativity, and domain-specific expertise. Proposition 4 therefore states that recognition will predict later domain competence more strongly when recognition is followed by sustained, appropriate, accessible developmental opportunities.

The third function is engagement and feedback. Self-determination theory identifies autonomy, perceived competence, and relatedness as basic psychological needs supporting motivation and well-being (Ryan and Deci, 2000; Deci and Ryan, 2000). Perceived competence is the experience of effectiveness, distinct from assessed domain competence. AI may support these needs through meaningful choice, challenge, and informative feedback, or undermine them through surveillance and dependence. A meta-analysis reported improvements in self-regulated and self-directed learning, with substantial variation across regulatory dimensions and contexts (Achuthan, 2025). Zhao (2026) reported improved self-regulation, engagement, persistence, and academic outcomes in a cluster-randomized e-mentoring trial with disadvantaged underachieving students, including a four-week follow-up. These findings motivate P5 but do not establish long-term talent development, effects in gifted populations, or superiority over equally resourced non-AI regulatory support.

The fourth function is developmental monitoring. Periodic identification and intervention assessments may miss changes between assessment occasions. Learning analytics can reveal rapid mastery, plateau, declining challenge, shifts in engagement, or persistent discrepancies between demonstrated competence and assigned work. A systematic review of learning analytics for study success found that analytics can support prediction and intervention but stressed the need for stronger pedagogical integration and attention to learner-facing support (Ifenthaler and Yau, 2020). Developmental monitoring in AI-LAMTD therefore differs from risk surveillance: it includes positive deviation, such as unexpectedly rapid growth or advanced transfer, as well as vulnerability. A high-achieving student whose engagement steadily declines may be experiencing developmental mismatch even while grades remain excellent.

The fifth function is support for well-being and flourishing. Proposed applications include making support resources more accessible, flagging patterns of overload or disengagement for human interpretation, and helping learners and educators reconsider goals, workload, and available support. These applications require population-specific validation and should preserve learner agency. Flourishing is evaluated through sustained competence together with well-being, agency, belonging, and meaningful contribution, rather than inferred from high achievement alone. Reviews of large language models in education identify possibilities for feedback and accessibility alongside risks involving hallucination, overreliance, privacy, and reduced critical engagement (Kasneci et al., 2023; Tlili et al., 2023; Lo, 2023). Bastani et al. (2025) show why immediate assisted output must be distinguished from later independent performance. Proposition 6 therefore tests whether retention and transfer without the focal AI assistance predict subsequent domain development more accurately than assisted achievement. Necessary accessibility accommodations should be retained and documented in such comparisons.

## 5 Theoretical foundations and architecture of AI-LAMTD

AI-LAMTD integrates several theoretical traditions by assigning each a distinct explanatory function. Developmental talent theories specify what develops: domain competence develops through sustained learning and practice, and may reach outstanding levels expressed in talent, expertise, and creative production (Gagné, 2004; Subotnik et al., 2011; Reis et al., 2021). Bioecological theory specifies where and through which recurring transactions development occurs, emphasizing person-process-context-time relations (Bronfenbrenner and Morris, 2006). The Actiotope Model specifies the adaptive learner-environment system through which goals, action repertoires, subjective action space, and environmental resources co-develop (Ziegler, 2005; Ziegler et al., 2013). Self-determination theory specifies a motivational conversion mechanism: opportunities are more likely to become sustained engagement when autonomy, perceived competence, and relatedness are supported (Ryan and Deci, 2000). The capability approach evaluates whether resources become substantive opportunities that learners can actually convert into valued functioning (Sen, 1999; Nussbaum, 2011). LAMTD organizes these processes across four ecological levels and foregrounds equity and capability expansion and cultural relevance and inclusion (Blumen, 2026, pp. 171–183).

These theories are complementary but not interchangeable. In this synthesis, talent-development theories guide domain progression; the capability approach directs attention to substantive opportunity and conversion conditions; self-determination theory informs motivation; and ecological and Actiotope accounts organize learner-environment relations. AI-LAMTD specifies how these explanatory roles connect within an architecture of recurrent conversion processes. This integration is useful only if it yields clearer operational distinctions and better predictions or interventions than simpler competing accounts.

The integration also creates tensions that AI-LAMTD treats as boundary conditions. Fixed or individual-capacity interpretations of giftedness can conflict with systemic accounts of development; algorithmic optimization can conflict with learner agency and culturally valued goals; predictive classification can reify provisional judgments; and personalization can narrow exploration when it repeatedly recommends what resembles prior performance. The model therefore requires revisable recognition, human oversight, learner contestability, culturally responsive validation, and outcomes that include independent capability, agency, and well-being.

AI is not an additional psychological theory. It becomes theoretically relevant when algorithmic systems participate in consequential information and allocation processes: determining which learners become visible, which opportunities are recommended, what challenge and feedback are supplied, how trajectories are monitored, and how learners relate to human and material resources. AI is neither necessary nor sufficient for talent development. Its inclusion in AI-LAMTD permits conditional predictions about how algorithmic mediation changes the timing, scale, and distribution of developmental transitions.

Within this architecture, LAMTD supplies four interacting ecological levels—individual development, learning contexts, family and community, and policy and infrastructure—and two cross-cutting principles: equity and capability expansion and cultural relevance and inclusion. Figure 1 links the complete set of developmental functions to all four levels. The links indicate possible cross-level operation, not a one-to-one allocation of functions to levels or evidence of equal effects at every level. Recognition may combine individual learning evidence, classroom observations, community knowledge, and institutional criteria; opportunity design may connect learner goals, pedagogy, mentors, and funded provision. AI may support these functions when its inferences can inform appropriate human decisions. Figure 2 specifies the developmental pathway and the recurrent processes to which such support may contribute.

**Figure 1:**
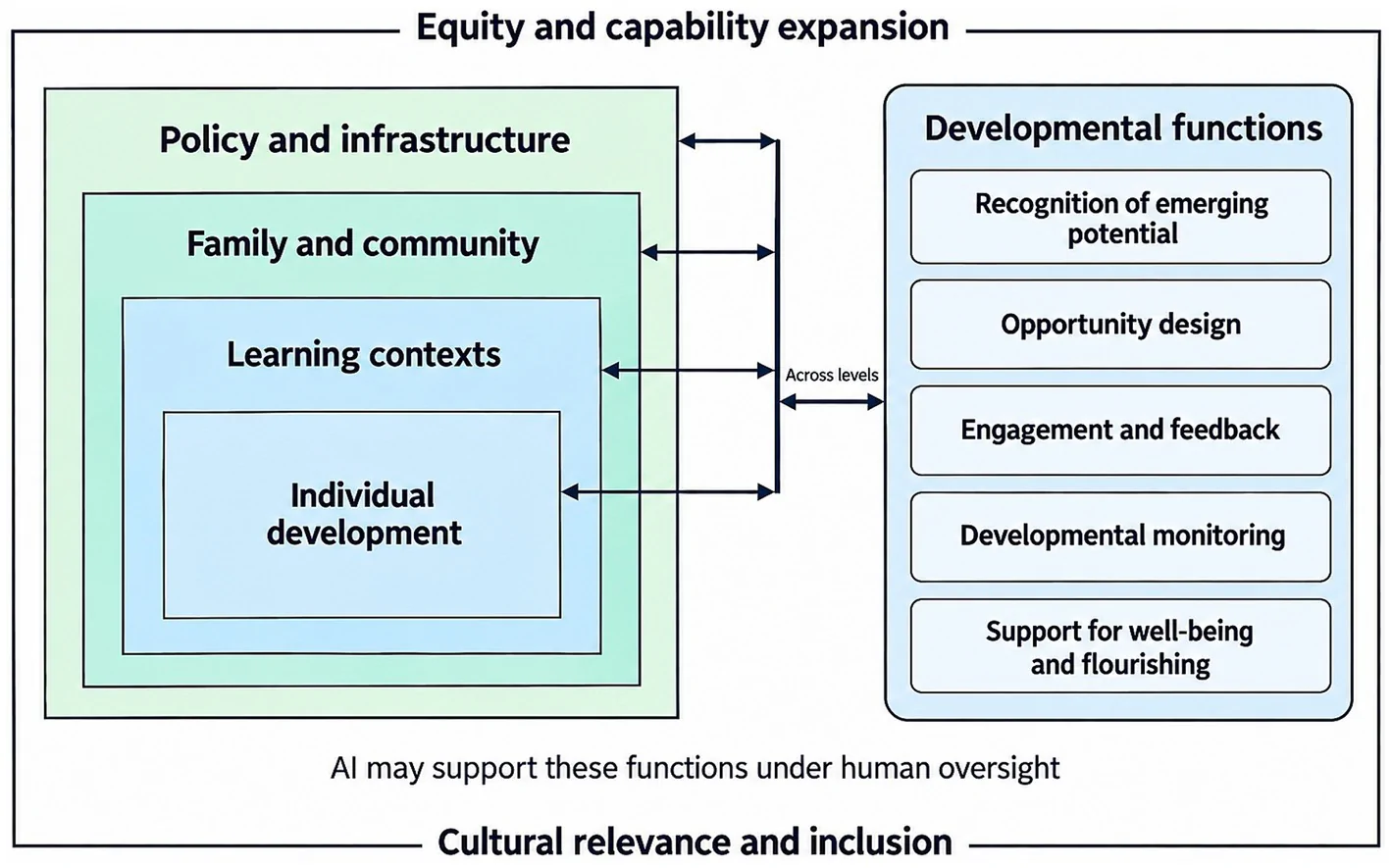
Ecological architecture of AI-LAMTD. Five developmental functions may operate across four ecological levels within two cross-cutting principles. Connections indicate possible cross-level relationships under human oversight, not fixed assignments or established causal effects.

**Figure 2:**
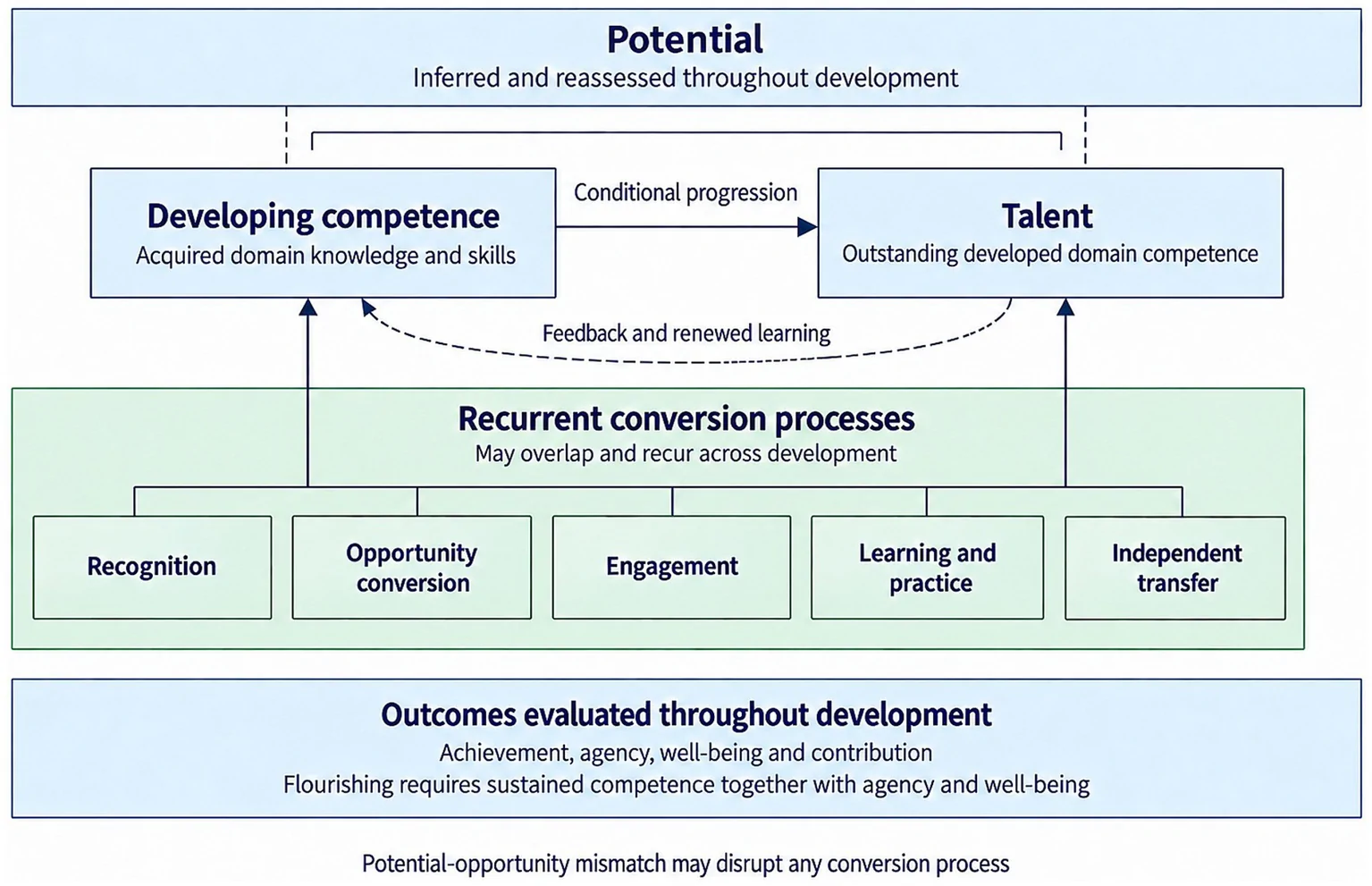
Developmental pathway in AI-LAMTD. Recurrent conversion processes support conditional progression from developing competence to talent. Potential is reassessed throughout; achievement, agency, well-being, and contribution are evaluated over time. Mismatch may disrupt any conversion process.

The 10 propositions define a conditional and falsifiable research program. Table 3 retains the focal constructs, decisive comparisons or conditions, and observations that would fail to support each prediction. Supplementary Table S1 provides units of analysis and illustrative study designs. General propositions can be tested without AI; AI-contingent versions test algorithmic implementation of a general mechanism; AI-specific propositions require comparison involving AI mediation. These classifications distinguish the technological condition from the psychological or educational mechanism. A study should prespecify its primary outcomes, meaningful effect thresholds, and decision rules; an imprecise null result alone cannot establish that a proposition has been refuted. Underachievement outcomes must be defined independently of the opportunity indicators used to explain them, with developmental expectations specified before follow-up.

**Table 3:** Core constructs and tests of the 10 AI-LAMTD propositions.

Proposition and class	Core constructs and predicted relationship	Comparison or condition	Observation that would fail to support the prediction
P1 ecological mismatch G	Learner-opportunity fit adds predictive value for longitudinal underachievement.	Comparable baseline potential; learner-only and discrepancy-only models.	Fit and opportunity indicators add no out-of-sample predictive value beyond either comparison model.
P2 dynamic recognition G/C	Dynamic, multidimensional evidence improves sensitivity for later advanced learning or production.	Achievement-dominant screening; prespecified specificity margin.	Sensitivity does not improve, or any gain violates the acceptable specificity margin.
P3 process indicators G/C	Learning rate, transfer, strategy change, and scaffold responsiveness add predictive value.	Static achievement model; varying prior-opportunity constraint.	Process indicators add no predictive value for later unassisted performance; the predicted larger gain under greater constraint is absent.
P4 opportunity conversion G	Access and uptake help explain the recognition-to-competence association.	Provision varies in access, challenge, and continuity.	Appropriate provision does not strengthen the association, or access and uptake do not mediate the predicted component.
P5 regulatory scaffolding A	Scaffolding improves delayed learning, transfer, and self-regulation.	Scaffolded AI versus answer-providing AI and a non-AI condition matched for goals, time, and resources.	Scaffolded AI shows no predicted advantage on prespecified outcomes relative to the comparison conditions.
P6 independent capability A	Unassisted retention and transfer predict later domain development better than assisted achievement.	Assisted and independent assessments on aligned occasions; delayed retention and transfer.	Assisted achievement predicts later development as accurately as or better than unassisted retention and transfer.
P7 capability expansion G/C	Reduced outcome disparities following improved recognition depend on accessible, sustained opportunity.	Recognition gains with improved versus persistently unequal conversion conditions.	Recognition alone reproducibly reduces downstream disparities despite persistently unequal conversion conditions, after accounting for alternative changes.
P8 cultural responsiveness A	Increasing cultural or linguistic distance disadvantages unadapted AI systems.	Locally validated or adapted versus unadapted systems across target contexts.	The predicted disadvantage of unadapted systems does not increase with distance on the prespecified validity, calibration, or error-rate measure.
P9 transition matching G	Support matched to the diagnosed conversion bottleneck improves relevant outcomes.	Generic intervention of comparable intensity; preregistered diagnosis.	Matched support does not outperform generic support on prespecified process-specific and downstream outcomes.
P10 sustainability G	Achievement, independent transfer, agency, and well-being jointly improve longitudinal prediction.	Achievement-only model; temporally separate follow-up criteria.	The multidimensional profile predicts later competence, engagement, and contribution no better than achievement alone.

G, general mechanism; C, AI-contingent implementation; A, AI-specific comparison. Criteria concern adequately powered tests with valid measures and prespecified decision rules. Supplementary Table S1 contains units of analysis and illustrative designs. Independent conditions remove the focal AI assistance while retaining necessary accessibility accommodations.

### 5.1 P1 ecological mismatch

Among learners with comparable baseline indicators of emerging potential, longitudinal underachievement will be predicted more accurately by learner-by-opportunity fit—including access, challenge, support, and continuity—than by learner characteristics or a cross-sectional potential-achievement discrepancy alone. *Mechanism class: General.*

### 5.2 P2 dynamic and multidimensional recognition

For learners whose prior opportunities are unequal or whose strengths and barriers may mask one another, a preregistered dynamic and multidimensional protocol will show higher sensitivity for later advanced learning or production than achievement-dominant screening, without an unacceptable loss of specificity. *Mechanism class: General; AI-contingent when automated.*

### 5.3 P3 learning-process indicators

Models adding learning rate, transfer, strategy change, and responsiveness to scaffolding will predict later unassisted advanced performance more accurately than otherwise equivalent models using static achievement alone, with the largest incremental validity expected under greater prior-opportunity constraint. *Mechanism class: General; AI-contingent when inferred algorithmically.*

### 5.4 P4 opportunity conversion

Recognition will predict later domain-specific competence more strongly when accessible, appropriately challenging, and sustained opportunities are offered and taken up; access and uptake will mediate part of the recognition-to-development association. *Mechanism class: General.*

### 5.5 P5 regulatory scaffolding

AI designed to scaffold goal setting, monitoring, strategic choice, reflection, and gradual fading will produce stronger delayed, transfer, and self-regulation outcomes than answer-providing AI matched for content exposure and than a non-AI comparison condition matched for instructional goals, contact time, and access to learning resources. *Mechanism class: AI-specific.*

### 5.6 P6 independent capability

AI-assisted performance will overestimate talent development when gains do not persist after assistance is withdrawn; unassisted retention and transfer will predict subsequent domain development more strongly than performance measured only with AI available. *Mechanism class: AI-specific.*

### 5.7 P7 equity through capability expansion

Improved recognition will reduce group disparities in developmental outcomes only when recommended opportunities are accessible, taken up, and sustained; where conversion conditions remain unequal, recognition gains alone will not reduce—and may widen—downstream disparities. *Mechanism class: General and AI-contingent.*

### 5.8 P8 cultural and linguistic responsiveness

As cultural and linguistic distance between model-development and target populations increases, unadapted AI systems will show poorer calibration, greater error-rate disparity, or lower construct validity than locally validated or culturally adapted systems. *Mechanism class: AI-specific.*

### 5.9 P9 transition-matched intervention

Among learners classified through a preregistered mismatch diagnosis, interventions matched to the disrupted transition will produce larger change on transition-specific and downstream outcomes than generic underachievement interventions of comparable intensity. *Mechanism class: General.*

### 5.10 P10 longitudinal developmental sustainability

A baseline profile combining achievement, independent transfer, agency, and well-being will predict later sustained competence, continued engagement, and meaningful contribution more accurately than an achievement-only model. Predictors and outcomes will be measured on separate occasions using prespecified criteria. *Mechanism class: General criterion; AI-LAMTD outcome test.*

AI-LAMTD organizes potential AI support through five developmental functions: recognition of emerging potential, opportunity design, engagement and feedback, developmental monitoring, and support for well-being and flourishing. These functions operate within the interacting levels of individual development, learning contexts, family and community, and policy and infrastructure. Functions describe what educational support is intended to accomplish; conversion processes describe how recognition, access, participation, learning, and transfer may produce developmental change. Their relationships are many-to-many: for example, opportunity design can support opportunity conversion, engagement, and learning and practice, while developmental monitoring can inform all five conversion processes. The functions may also be implemented without AI.

At the level of individual development, relevant processes include cognitive and domain-specific abilities, creativity, curiosity, interests, motivation, identity, self-regulation, executive functioning, socioemotional processes, and well-being. AI can contribute through dynamic assessment, learner modeling, personalized challenge, metacognitive prompts, and longitudinal pattern recognition. However, a learner model must remain provisional. The person is not the profile, and algorithmic inference must not become an identity assignment.

This position is consistent with Ouyang and Jiao’s (2021) distinction among AI-led, AI-supported, and AI-empowered paradigms, with more human-centered applications positioning learners as active collaborators and agents in their learning.

At the level of learning contexts, teachers, curricula, pedagogy, assessment, peers, mentors, and educational technologies shape whether recognized potential is met with developmentally appropriate challenge. The model calls for dual differentiation: simultaneously addressing barriers and extending strengths. This is especially relevant for 2e learners, who may require assistive technology or explicit executive-function support while also needing acceleration or advanced conceptual challenge (Beckmann and Minnaert, 2018; Townend et al., 2024). This position is consistent with enrichment research showing the value of flexible identification and appropriately challenging learning opportunities for students with diverse profiles of potential (Reis and Renzulli, 2010; Reis and Peters, 2021).

Family and community constitute the third ecological level. Families and communities provide emotional support, expectations, cultural meaning, language, knowledge traditions, social networks, resources, and access to opportunity. AI may reduce geographical isolation by connecting learners with experts, communities, specialized courses, and advanced resources that are not available locally. However, access to devices, connectivity, digital literacy, quiet study space, time, and adult support remains uneven. OECD (2021) emphasizes that digital transformation in education depends on capacity, governance, infrastructure, and inclusion. In AI-LAMTD, digital access is therefore not a single variable but a conversion factor: the same technological resource can yield very different developmental opportunities across ecosystems.

Policy and infrastructure form the outer layer. Identification rules, funding, teacher education, admissions pathways, public infrastructure, data governance, and institutional capacity help determine whether individual recommendations can become real opportunities. AI cannot compensate for the absence of appropriate programs or for poor educational provision. A model may identify advanced potential accurately, but if no appropriate challenge, mentor, or institutional pathway follows, recognition does not become development. Likewise, a platform that works well in well-resourced schools but is unavailable elsewhere can widen rather than narrow opportunity gaps.

Two principles cut across all four levels. Equity and capability expansion require asking who becomes visible, who gains access to meaningful developmental opportunities, who can act on recommendations, and who bears the consequences of error. Data and design must therefore be responsive to learners’ languages, knowledge systems, identities, and culturally situated forms of achievement. Culturally sustaining pedagogy argues that education should sustain rather than erase plural cultural and linguistic practices (Paris, 2012), a particularly important consideration for AI systems trained predominantly on high-resource languages and datasets. UNESCO’s Recommendation on the Ethics of Artificial Intelligence, adopted in 2021, likewise emphasizes human rights, diversity, fairness, transparency, and human oversight (UNESCO, 2022). Its Guidance for Generative AI in Education and Research provides a human-centered, age-appropriate, and equitable framework for AI use (Miao and Holmes, 2023). The AI Competency Framework for Teachers similarly emphasizes professional judgment in relation to ethics, pedagogy, and human agency (Miao and Cukurova, 2024).

These principles also argue against opaque predictive models. Comparative analyses of AI ethics guidelines have identified transparency, justice and fairness, non-maleficence, responsibility, and privacy as recurring principles (Jobin et al., 2019). Holmes et al. (2022) further argue that ethical AI in education requires community-wide attention to agency, fairness, pedagogy, accountability, and the values embedded in system design. Work on algorithmic systems also warns that optimization can obscure structural inequities and convert contested social judgments into apparently objective scores (O’Neil, 2016). AI-LAMTD therefore treats explainability, contestability, and human-in-the-loop interpretation as developmental requirements rather than optional ethical additions.

## 6 Developmental pathway and recurrent conversion processes

Figure 2 distinguishes developmental levels, ongoing inference, conversion processes, and evaluated outcomes. The central trajectory represents developing domain competence and its possible progression to talent, defined here as outstanding developed domain competence. Progression is conditional, domain-specific, and open to renewed learning. Potential is inferred and reassessed throughout this trajectory, including when talent is already evident. Giftedness or high ability describes exceptional capacity relative to a specified reference context; it is not an additional stage through which every learner must pass. Achievement records particular accomplishments at different points along the trajectory.

Five conversion processes may overlap, recur, and operate at different developmental levels. Recognition makes emerging or developed strengths visible through revisable judgments. Opportunity conversion turns available resources and recommendations into accessible provision that learners can take up and sustain. Engagement involves meaningful participation supported by learner goals, autonomy, perceived competence, and relatedness. Learning and practice concern the activities through which domain knowledge and skills are acquired, consolidated, and reorganized. Independent transfer involves applying and adapting what has been learned beyond the focal assistance and original task context. These processes have practical dependencies within particular episodes, but the model specifies no universal order: learning can reveal unrecognized strengths, new opportunities can follow achievement, and transfer evidence can prompt renewed recognition and revised provision.

The processes locate testable bottlenecks rather than define a second developmental pathway. P2 and P3 test the evidence used in recognition; P4 and P7 examine whether provision becomes accessible, sustained opportunity; P5 tests regulatory support for engagement and learning and practice; and P6 tests the distinction between assisted achievement and independent retention and transfer. P1 and P9 examine fit and process-specific intervention, while P8 examines cultural and linguistic validity. A learner may encounter several bottlenecks simultaneously or return to an earlier problem when tasks, goals, or circumstances change. The feedback arrow in Figure 2 represents reassessment and renewed learning, rather than an inevitable reversal or a fixed sequence of stages.

Achievement, agency, well-being, and contribution are evaluated throughout development. Flourishing describes sustained competence that coexists with agency, well-being, belonging, and meaningful contribution; it is a multidimensional evaluative outcome rather than an obligatory stage after talent. P10 tests whether a baseline profile combining achievement, independent transfer, agency, and well-being predicts later sustained competence, engagement, and contribution more accurately than achievement alone. Baseline predictors and follow-up outcomes must be temporally separate, with independent tasks or criteria where appropriate, to avoid defining success through the same observations used to predict it.

## 7 Human centered design principles and empirical research agenda

AI-LAMTD is guided by six design principles: augmentation of human judgment and learning; strengths-sensitive evidence; dynamic and revisable inference; explainability, contestability, and correction; cultural and linguistic responsiveness; and preservation of learner agency and well-being. These principles are consistent with guidance emphasizing human oversight, inclusion, professional competence, rights, and accountability in educational AI (UNESCO, 2022; Miao and Holmes, 2023; Miao and Cukurova, 2024). They are normative commitments; their proposed effects on trust, engagement, opportunity conversion, and development require empirical testing.

Predictive accuracy and average progress are insufficient evaluation criteria. A human-in-the-loop system must allow educators, psychologists, learners, and families to interpret evidence in context and to challenge an inference or recommendation. A detected decline in engagement, for example, may reflect boredom, anxiety, family stress, identity conflict, inadequate challenge, cognitive overload, disability-related barriers, or a deliberate change in domain interest. The same algorithmic signal can therefore require different actions, including no intervention. Human oversight must include authority to revise classifications, redirect recommendations, protect privacy, and stop a system that produces harm.

Learners must also be active participants in the process. Personalization should help them understand relevant evidence, formulate goals, ask questions, and co-design developmental trajectories. Holmes et al. (2022) identify agency and accountability as core ethical issues in AI in education, while Ouyang and Jiao’s (2021) AI-empowered paradigm similarly positions the learner in a more active role.

Future research should evaluate AI-LAMTD through longitudinal, experimental, quasi-experimental, mixed-method, and participatory designs. Recognition studies should compare static screening with learning trajectories and dynamic indicators across socioeconomic, linguistic, geographic, gender, and disability groups. Experimental studies should distinguish performance with AI from delayed performance without AI and include transfer, self-regulation, metacognition, and agency as outcomes. Comparisons should align content exposure, task difficulty, assessment timing, and human support so that advantages are not attributable to unequal provision. Equity studies should audit the entire pathway, from recognition and recommendation through access, participation, development, and outcomes, because an algorithm may be statistically fair at the classification stage while the broader system remains developmentally inequitable.

Twice-exceptional research is particularly important because 2e profiles challenge traditional learner models. Future studies should examine whether separate modeling of strengths and barriers improves both gifted identification and accommodation decisions relative to global achievement or ability indices. International and cross-cultural validation should evaluate how models transfer across languages and educational systems and where local calibration is required. Participatory studies should involve students, teachers, psychologists, families, and communities in determining what constitutes meaningful talent development, acceptable AI support, and culturally valid evidence. At the same time, the emerging evidence base should not be interpreted too optimistically or used to justify technological solutionism. Zawacki-Richter et al. (2019) found that educators were underrepresented in much early AIEd research, while Chen et al. (2020) documented both promising educational applications and substantial implementation challenges. In gifted education, Alsamani and Alsamiri (2026) similarly conclude that AI research is promising but still at an early stage, with major gaps in longitudinal outcomes, twice-exceptional education, and equity. These limitations strengthen the case for theory-driven research.

The research agenda should treat model failure as informative. Equal recognition with unequal opportunity uptake directs investigation toward opportunity conversion and institutional capacity. High participation with weak retention or independent transfer directs attention to learning and practice, the quality of scaffolding, and transfer conditions. Rising achievement with declining well-being calls for scrutiny of workload, agency, and support. These patterns guide competing hypotheses; establishing their causes requires appropriate comparison conditions and attention to measurement error, learner goals, and contextual change.

### 7.1 Boundary and failure conditions

AI-LAMTD does not predict positive outcomes merely from technology adoption. Positive effects are unlikely when institutions cannot act on recommendations; infrastructure, devices, connectivity, or digital literacy are insufficient; training and validation data do not represent target populations; outcome targets privilege narrow performance; surveillance undermines privacy, trust, or autonomy; language and cultural assumptions are transferred without local validation; AI substitutes for cognitive or regulatory work; or professional oversight, explanation, appeal, and correction are absent.

These conditions are moderators within the theory rather than external implementation details. The same capability may expand opportunity in a well-supported ecology, produce no developmental change where provision is absent, or widen inequality when access and institutional conversion capacity are stratified. Failure should be investigated at the level of the relevant conversion process. Equal recognition with unequal uptake is consistent with an opportunity-conversion bottleneck; high participation with weak independent transfer is consistent with difficulties in learning and practice or transfer; achievement accompanied by deteriorating well-being indicates that outcome evaluation must extend beyond accomplishment alone.

## 8 Discussion

AI-LAMTD explains underachievement through the changing relation between learners and their developmental ecology. Its proposed advance over LAMTD is to specify recurrent conversion processes whose operation can be compared across levels and over time. The distinction among potential, high ability, competence, talent, and achievement separates inference about future development, comparative capacity, acquired skill, distinguished domain competence, and observed accomplishment. This separation makes it possible to test whether learners with similar achievement discrepancies face different bottlenecks, including inaccurate recognition, inaccessible opportunity, weak engagement, inadequate support for learning and practice, or limited independent transfer. The claimed explanatory value depends on whether these distinctions improve prediction and intervention relative to simpler accounts.

The framework also changes the meaning of personalization. Adaptation must consider whether work is sufficiently challenging, whether strengths are extended, whether barriers suppress performance, and whether competence persists beyond assistance. General AIEd findings support the plausibility of adaptive and regulatory mechanisms, but they do not establish equivalent effects in gifted education. Direct evidence for gifted, high-ability, and twice-exceptional populations remains limited and should be evaluated through longitudinal, comparative, and equity-focused designs (Alsamani and Alsamiri, 2026).

The Latin American designation identifies the model’s theoretical origin and epistemic standpoint. Regional evidence concerning unequal learning conditions, digital access, multilingualism, rurality, and heterogeneous institutional provision illustrates why opportunity and conversion capacity cannot be assumed constant (UNESCO Regional Office for Education in Latin America and the Caribbean, 2021; Economic Commission for Latin America and the Caribbean, 2022; Blumen, 2026). The framework does not presume that Latin America is homogeneous or that mechanisms demonstrated in one country generalize automatically. Cross-national and within-country variation supplies the contrasts needed to test which transition mechanisms are robust, locally contingent, or measurement dependent.

### 8.1 Competing explanations remain possible

Apparent recognition mismatch may reflect measurement error rather than suppressed potential; nonparticipation may reflect an autonomous change in valued goals rather than engagement failure; developmental gains may arise from teacher quality, peer networks, maturation, or family support rather than AI mediation; and algorithmic recommendations may proxy institutional advantage. Tests of AI-LAMTD should therefore include plausible rival predictors, baseline trajectories, non-AI comparison conditions where appropriate, and qualitative evidence about learner meaning and choice.

The model’s boundary conditions are substantive. Positive effects are unlikely when institutions cannot act on recommendations; infrastructure, connectivity, devices, or digital literacy are inadequate; training and validation data do not represent target populations; target variables privilege narrow achievement; surveillance undermines privacy, trust, or autonomy; language and cultural assumptions are transferred without local validation; AI substitutes for cognitive or regulatory work; or explanation, appeal, correction, and professional oversight are absent. Improved recognition can itself produce harm when it raises expectations, but no viable developmental pathway follows.

AI-supported approaches may also fail through overdependence and displacement. Systems that optimize immediate output can weaken independent practice, narrow exploration, or transfer authority from learners and educators to opaque recommendations. Even a technically accurate model may widen inequality if affluent learners can act on recommendations while others cannot. Equity must therefore be evaluated across the full chain from recognition and recommendation to access, uptake, continuity, independent development, agency, well-being, and contribution.

### 8.2 Several limitations remain

AI-LAMTD is a theoretical framework requiring tests across ages, domains, languages, countries, and educational systems. Potential, flourishing, cultural relevance, and agency require careful and locally validated operationalization. Dynamic data can become intrusive when collection exceeds pedagogical need. Causal claims require experimental or strong quasi-experimental designs, whereas flourishing and contribution require follow-up periods longer than most educational technology studies provide. The framework may also become overly complex if every contextual factor is treated as a moderator. Empirical work should therefore test parsimonious process-specific models before attempting complete system estimation. Model complexity should be evaluated with held-out prediction and prespecified outcomes, rather than judged from improved fit to the same data used to develop the model.

These limitations make failure informative. A process-specific explanation is weakened when it fails to improve prediction or intervention beyond learner-only, discrepancy-only, or other plausible competing models. Improved recognition without improved access is compatible with P7’s conditional prediction and is not, by itself, evidence against it. Conversely, reproducible reductions in downstream disparities attributable to recognition alone, despite persistently unequal conversion conditions, would challenge that prediction. Across propositions, tests should distinguish a failure of a particular intervention, inadequate measurement, and evidence that the proposed relationship itself is unsupported.

## 9 Conclusion

AI-LAMTD distinguishes potential as a revisable inference, giftedness or high ability as a context-relative description of exceptional capacity, competence as acquired domain knowledge and skills, talent as outstanding developed competence, and achievement as observable accomplishment. Recognition, opportunity conversion, engagement, learning and practice, and independent transfer may overlap and recur as competence develops. Potential remains open to reassessment, and flourishing requires evidence of sustainable development together with agency and well-being. Underachievement may reflect disruption of different conversion processes, so similar achievement gaps may call for different explanations and interventions.

AI capabilities may improve visibility, adaptation, feedback, monitoring, mentoring, and resource matching. Their developmental value depends on the technology used, the validity of its inferences, the pedagogy in which it is embedded, and whether ecological systems convert information into accessible and culturally relevant opportunity. The framework predicts no benefit, and possible harm, when data are biased, access is stratified, recommendations cannot be acted upon, surveillance displaces trust, or AI substitutes for learning and human judgment.

AI-LAMTD thus offers a conditional and falsifiable account of talent development in increasingly algorithmically mediated ecologies. Its central criterion is not immediate AI-assisted performance, but sustained competence, transfer, agency, well-being, continued engagement, and meaningful contribution.
