# Supplementary material for: Bridging potential and achievement: the AI-enhanced Latin American model of talent development
Source: Front Psychol. 2026 Oct 14;17:1988238. doi: 10.3389/fpsyg.2026.1988238 (PMC13713650; doi:10.3389/fpsyg.2026.1988238)
Supplement: Supplementary file 1 [file Table_1.docx]

Supplementary methods for testing AI-LAMTD

Companion to Bridging Potential and Achievement: The AI-Enhanced Latin American Model of Talent Development. This supplement retains illustrative research designs and units of analysis; the predictions and non-support criteria are reported in main-text Table 2. These are proposed studies, not completed empirical validations.

# Table S1. Units of analysis and illustrative study designs

| **Proposition** | **Unit of analysis** | **Illustrative design** | **Measurement and comparison priorities** |
| --- | --- | --- | --- |
| P1 | Learner in context | Longitudinal multilevel or person-context interaction model | Measure baseline learning indicators and opportunity conditions separately. Define the follow-up underachievement outcome independently of opportunity predictors. Compare held-out prediction against learner-only and discrepancy-only models. |
| P2 | Learner and identification decision | Diagnostic-accuracy study with subgroup sensitivity and false-negative analysis | Prespecify the reference population, identification threshold, sensitivity target, and acceptable specificity loss. Use later advanced learning or production as an independent criterion. |
| P3 | Learning episode and learner | Incremental-validity study with cross-validated predictive modeling | Compare nested models using the same validation data. Test moderation by prior-opportunity constraint; prevent leakage from outcomes into process indicators. |
| P4 | Learner, program, and institution | Longitudinal mediation, natural experiment, or propensity-score design | Separate offered access, actual uptake, challenge, and continuity. Assess confounding and time order before interpreting mediation causally. |
| P5 | Learner and task | Randomized experiment with scaffold fading and delayed posttest | Match instructional goals, content exposure, contact time, and resource access across AI and non-AI conditions. Prespecify scaffold fading, delayed transfer tasks, and self-regulation measures. |
| P6 | Learner across occasions | Crossover or longitudinal withdrawal design | Compare assisted and independent assessments on equivalent tasks and aligned occasions; test delayed retention and transfer. Retain access accommodations and predict later competence using separate criteria. Address order and carryover effects. |
| P7 | Learner, institution, and system | Pathway equity audit, multigroup mediation, or implementation study | Compare improved recognition with and without improved conversion conditions. Continued inequality after recognition alone is compatible with P7; test whether access and uptake explain downstream changes. |
| P8 | Model, learner group, and site | Cross-cultural validation, measurement-invariance study, or fairness audit | Specify cultural and linguistic distance independently of the outcome. Select primary validity, calibration, or error-rate measures and compare adapted and unadapted systems across contexts. |
| P9 | Learner and intervention | Stratified randomized or quasi-experimental comparative study | Preregister a reproducible conversion-bottleneck diagnosis. Match intervention intensity and examine process-specific and downstream outcomes, including rival explanations. |
| P10 | Learner across time | Prospective longitudinal study with preregistered independent criteria | Measure achievement, independent transfer, agency, and well-being at baseline. Assess competence, engagement, and contribution later using independent tasks or criteria; compare out-of-sample prediction. |

Note. Designs are illustrative and require adaptation to the domain, population, and local context. Primary outcomes, effect thresholds, uncertainty, subgroup analyses, and decision rules should be specified in advance. Failure to reject a null hypothesis in an underpowered or poorly measured study is not equivalent to evidence against a proposition.
